# High-Performance Ternary NiCoMo Electrocatalyst with Three-Dimensional Nanosheets Array Structure

**DOI:** 10.3390/nano12213716

**Published:** 2022-10-22

**Authors:** Zhihao Zhou, Zhi Lu, Shilin Li, Yiting Li, Gongliang Tan, Yang Hao, Yu Wang, Yuzhao Huang, Xuefeng Zhang, Shuaifang Li, Chong Chen, Guangxin Wang

**Affiliations:** 1 School of Materials Science and Engineering, Henan University of Science and Technology, Luoyang 471003, China; 2 Luoyang Key Laboratory of High Purity Materials and Sputtering Targets, Henan University of Science and Technology, Luoyang 471003, China; 3 Fonlink Photoelectric (Luo Yang) Co., Ltd., Luoyang 471000, China

**Keywords:** layered double hydroxide, electrocatalysis, oxygen evolution reaction, nanosheets

## Abstract

Oxygen evolution reaction is a key process in hydrogen production from water splitting. The development of non-noble metal electrode materials with high efficiency and low cost has become the key factor for large-scale hydrogen production. Binary NiCo-layered double hydroxide (LDH) has been used as a non-noble metal electrocatalyst for OER, but its overpotential is still large. The microstructure of the catalyst is tuned by doping Mo ions into the NiCo-LDH/NF nanowires to form ternary NiCoMo-LDH/NF nanosheet catalysts for the purpose of enhancing the active sites and reducing the initial overpotential. Only 1.5 V (vs. reversible hydrogen electrode (RHE), ≈270 mV overpotential) is required to achieve a catalytic current density of 10 mA cm^−2^ and a small Tafel slope of 81.46 mV dec^−1^ in 1 M KOH solution, which manifests the best performance of NiCo-based catalysts reported up to now. Electrochemical analysis and micro-morphology show that the high catalytic activity of NiCoMo-LDH/NF is attributable to the change of the microstructure. The interconnected nanosheet arrays have the obvious advantages of electrolyte diffusion and ion migration. Thus, the active sites of catalysts are significantly increased, which facilitates the adsorption and desorption of intermediates. We conclude that NiCoMo-LDH/NF is a promising electrode material for its low cost and excellent electrocatalytic properties.

## 1. Introduction

With the rapid development of the economy and society, the excessive consumption of fossil fuels has aggravated global energy and ecological crises, so the demand for clean energy has become urgent [1,2,3]. As a clean and efficient energy carrier, hydrogen energy has been widely considered to be a most promising energy source to replace oil and gas [4,5]. The device for converting electrolytic water to hydrogen is simple, easy to operate and gives high-purity hydrogen, and thus a most promising technology for clean hydrogen production. Water splitting involves two half-reactions, the OER and the HER [6]. However, water splitting efficiency needs to be improved through electrocatalysts. At present, the noble metal catalysts, such as RuO_2_, IrO_2_ and Pt show high catalytic activity in OER and HER, but their high cost seriously hinders their large-scale application [7].

In recent years, transition-metal-layered double hydroxides (LDHs) have shown great potential in the field of electrocatalytic hydrogen and oxygen precipitation [8,9]. Transition metal catalysts have received more and more attention because of their high reserves, low prices, good catalytic activity and stability. The surface engineering treatment of LDH-based electrocatalysts, including heteroatom incorporation and construction defects, can effectively modify the electron distribution of adjacent catalytic atom centers, which contributes to improving the intrinsic catalytic activity of catalytic sites and the electrocatalytic performance of the catalyst [10,11,12].

At present, several layered double hydroxide catalysts have been reported, such as NiFe-LDH, NiCo-LDH and NiAl-LDH electrocatalysts [13,14,15,16,17]. Compared with binary alloys, ternary alloy nanoparticles show the best catalytic activity in HER and OER. Li et al. used a one-step hydrothermal method to prepare vanadium-doped three-component NiFeV-layered double hydroxides on nickel foam (NF). The electronic structure of the catalyst was adjusted, the band gap was shorted, making the electron transfer easier, the active sites were enriched and the efficiency of electrocatalytic oxygen evolution was improved. In 1 M KOH, the overpotential was 195 mV and the Tafel slope was 42 mV dec^−1^ [18]. Long et al. synthesized an LDH that contained three metals (Fe, Ni and Co) as an advanced electrocatalyst by controlling the proportion of trivalent ions. With increasing Co content, the thickness of the LDH decreased, and the FeNiCo LDHs showed improved OER activity. The number of active sites was increased, which was attributed to the addition of Co, which can modulate the electronic structure of the catalytic center [19]. Jiang et al. prepared a high-performance electrocatalytic hydrolysis electrode by growing NiCo-LDH nanosheet arrays on the surface of NF, and changing the total concentration of metal ions. Attributed to its inherent lamellar structure and unique redox characteristics, the potential overpotential in alkaline medium was as low as 290 mV, which can be compared to a RuO_2_ catalyst. It was also superior to a Pt/C catalyst due to the excellent OER activity [20]. Lin et al. found that the adsorption energy barrier of Co to H was low according to theoretical calculations. The electrochemical test results showed that after a long electrocatalytic cycle, the stability of the electrode catalyst became poor due to the dissolution of alloy and grain agglomeration [21,22]. Mo cations have excellent adsorption performance for hydrogen. The introduction of Mo^6+^ can effectively improve the hydrolysis process. The introduction of Mo cation into NiCo-LDH catalysts can obtain a bifunctional catalyst. As have been reported, that showed the best catalytic activity in HER and OER, but the mechanism was not clear [23,24,25]. In addition, the catalytic performance of transition-metal-based catalysts can be improved using 3D porous nanostructures such as nickel foam, carbon cloth and ordered nanostructures [26,27]. The 3D porous structure is applied to the electrode catalyst as the substrate material, and the combination of the primary pores of the substrate material and the secondary pores of the catalyst improves the active area of the electrocatalyst, which makes the electrolyte and the catalyst fully contact, and is beneficial to the improvement of the catalytic activity and the adsorption and desorption of intermediates.

Herein, we prepare a series of Mo-doped NiCo-LDH nanosheet catalysts on NF substrate via a one-step hydrothermal method for OER electrocatalysis. The NiCoMo-LDH nanosheets were grown in situ on NF with porous structure. The in situ synthesis can ensure the electrolyte and catalyst fully contact, which can improve the activity of the catalyst. The Mo-doped NiCo-LDH/NF shows excellent OER performance. Compared with noble metal catalysts, the NiCoMo-LDHs exhibit not only a higher electrocatalytic activity, but also a low cost. They are expected to show promising applications in hydrogen production.

## 2. Materials and Methods

### 2.1. Materials

Nickel foam (99.9% purity, thickness of 1.5 mm, porosity of 97%), Nickel(II) nitrate hexahydrate (Ni(NO_3_)_2_·6H_2_O, 98% purity), Cobalt(II) nitrate hexahydrate (Co(NO_3_)_2_·6H_2_O, 98% purity) and Sodium molybdate(VI) dihydrate (NaMoO_4_·2H_2_O, 99% purity) were purchased from Shanghai Aladdin Biochemical Technology Company. (Shanghai, China). Urea (CO(NH_2_)_2_) and potassium hydroxide (KOH) were purchased from Sinopharm group. (Shanghai, China). All chemicals were analytical grade and used directly without any further purification. The test water was deionized water.

### 2.2. Synthesis of Nickel-Cobalt-Molybdenum Layered Double Hydroxide Materials

**Figure 1:**
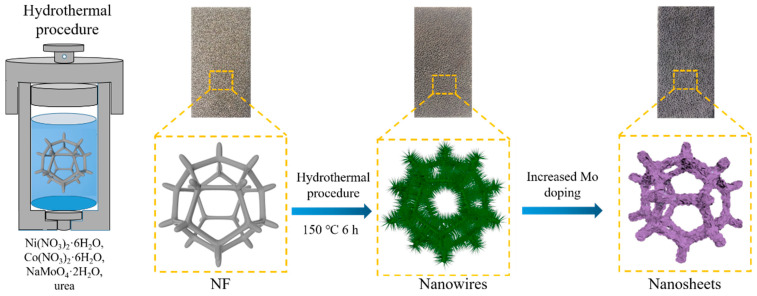
Schematic illustration for the one-step fabrication route of NiCoMo-LDH nanosheet arrays on NF.

The NiCoMo-LDH/NF electrode was synthesized by a one-step hydrothermal method, as shown in Figure 1. The NF (3 cm × 3 cm) was immersed in 3 M HCl solution for ultrasonic treatment for 15 min to remove the surface oxide, and then washed in ethanol and deionized water in turn several times and dried in a vacuum drying oven. In a typical process, 4 mmol Ni(NO_3_)_2_·6H_2_O, 4 mmol Co(NO_3_)_2_·6H_2_O and 2 mmol NaMoO_4_·2H_2_O were dissolved in 50 mL distilled water. Then, 12 mmol urea was added to the above solution with constant stirring. The mixture was transferred to a 100 mL Teflon-lined stainless steel autoclave and heated at 150 °C for 6 h. After cooling to room temperature, the as-synthesized samples were washed with ethanol and deionized water several times and dried at 60 °C overnight in a vacuum drying oven. In order to optimize the catalytic performance of electrode materials, the NiCoMo-LDH/NF catalyst was synthesized with several concentrations of molybdate sources (0 mmol, 1 mmol, 1.5 mmol, 2 mmol, 2.5 mmol and 3 mmol), and the corresponding samples were denoted as NiCo-LDH/NF, NiCoMo_1_-LDH/NF, NiCoMo_1.5_-LDH/NF, NiCoMo_2_-LDH/NF, NiCoMo_2.5_-LDH/NF and NiCoMo_3_-LDH/NF, respectively.

### 2.3. Characterization

X-ray diffractometer (Bruker-AXS D8 Advance, BRUKER, Ltd., Kyoto, Japan) and transmission electron microscopy (TEM, JEM-2100, JEOL Ltd., Tokyo, Japan) were used to characterize the phase structure and the crystal structure of NiCoMo-LDH/NF. The surface morphology and elemental analysis of NiCoMo-LDH/NF were observed by field emission electron microscopy and energy dispersive spectroscopy (FESEM, JSM-7800F, JEOL Ltd., Tokyo, Japan).

### 2.4. Electrochemical Measurements

The catalyst performance was tested in a standard three-electrode system by using a CHI660E electrochemical workstation (CH Instruments, Shanghai, China). The catalyst-loaded nickel foam was cut into a working electrode with a size of 0.5 cm × 1 cm, the counter electrode was a Pt plate (1 cm × 1 cm), and the reference electrode was an Ag/AgCl electrode filled with saturated KCl solution. All potential was converted to the reversible hydrogen electrode (RHE) scale according to the Nernst equation: *E*_RHE_ = *E*_Ag/AgCl_ + 0.1989 V + 0.059 × pH. In order to ensure that the inside of the electrolyte reached oxygen saturation during the electrochemical test, it was necessary to degas the electrolyte with oxygen for 30 min before the test. Linear sweep voltammetry (LSV) curves of OER of electrocatalysts were measured in 1.0 M KOH solution (pH = 13.7) at a scan rate of 2 mV s^−1^. Before electrochemical testing, iR compensation was performed on all LSV curves to eliminate the test error between the reference electrode and the working electrode. The same three electrodes with AC impedance were used for electrochemical impedance spectroscopy (EIS) in the frequency range between 0.01 and 104 Hz. With multiple scan speeds ranging from 1 to 5 mV/s, CVs were used to determine the electrochemical surface area (ECSA). In this paper, overpotential is determined using the formula η(V) = *E*_RHE_ − 1.23.

## 3. Results and Discussion

### 3.1. Structure and Morphology Characterization of NiCoMo-LDH/NF Catalyst

**Figure 2:**
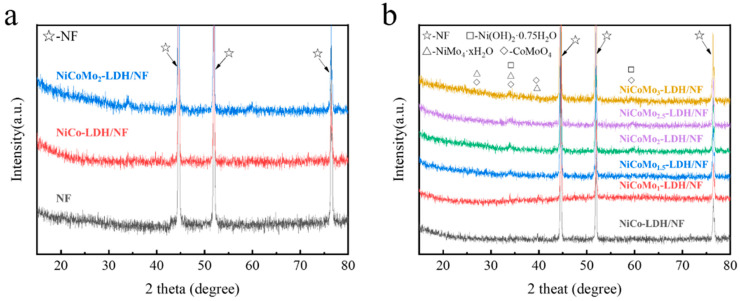
(**a**,**b**) XRD patterns of the as-prepared samples.

The crystal structure of the samples was studied by X-ray diffraction. As shown in Figure 2, there are obvious diffraction peaks in the sample of the supported catalyst, and the diffraction peaks correspond to Ni(OH)_2_ (JCPDF No.03-0177), NiMo_4_·xH_2_O (JCPDF No.13-0128) and CoMoO_4_ (JCPDF No.21-0868) [28,29], respectively. So, the NiCoMo-LDH catalyst was successfully prepared and loaded onto the NF substrate.

**Figure 3:**
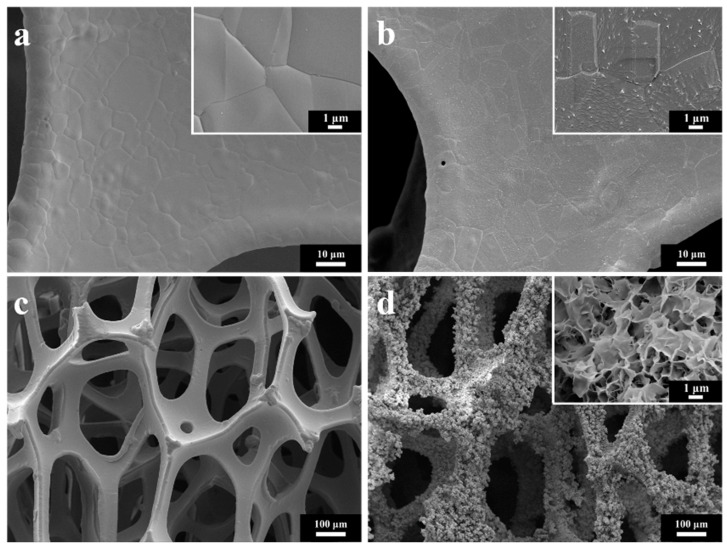
Low magnification and high magnification inset SEM images of (**a**) NF before pretreatment, (**b**) NF after pretreatment, (**c**) NF before hydrothermal treatment and (**d**) Low magnification and high magnification inset SEM images of NiCoMo_2_-LDH/NF after hydrothermal treatment.

The low- and high-magnification SEM images of the NF surface before pretreatment are shown in Figure 3a–c. It shows that before pretreatment the surface of the NF is smooth and covered by a dense oxide layer. To the pretreated NF (Figure 3b), the acid washing destroyed the surface oxide layer and made the surface of the NF rougher, which is favorable to the growth of the catalyst. As shown in Figure 3c, the NF has a three-dimensional network and macroscopic porous structure. The surface of the NiCoMo-LDH catalyst-loaded NF grew uniformly on the NF substrate as shown in Figure 3d. The combination of the primary pores of nickel foam and the secondary pores of nanosheet are not only good for improving the charge transfer efficiency, but also for increasing the active surface area of the catalyst.

**Figure 4:**
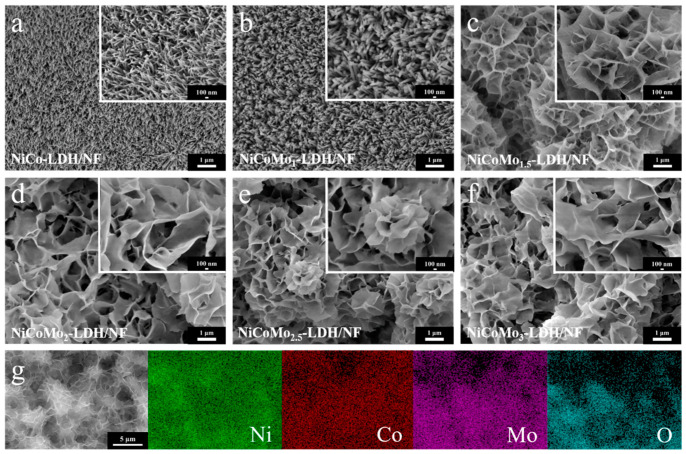
Low magnification and high magnification inset SEM images of (**a**–**f**) NiCo-LDH/NF, NiCoMo_1_-LDH/NF, NiCoMo_1.5_-LDH/NF, NiCoMo_2_-LDH/NF, NiCoMo_2.5_-LDH/NF and NiCoMo_3_-LDH/NF, respectively, and (**g**) EDS of NiCoMo_2_-LDH/NF nanosheets.

The morphologies of the as-prepared samples were observed through FESEM. The morphological characteristics of different Mo-doped electrocatalysts are shown in Figure 4. As shown in Figure 4a, NiCo-LDH/NF has a dense and fine nanowire structure. With increasing Mo content, the nanowires are gradually coarsened to form a NiCoMo_1_-LDH/NF nanorod structure (Figure 4b) and a NiCoMo_1.5_-LDH/NF lamellar linear structure (Figure 4c). NiCoMo_2_-LDH/NF, NiCoMo_2.5_-LDH/NF and NiCoMo_3_-LDH/NF show flower-like morphologies due to the continued increase of Mo (Figure 4d–f). Compared with the nanowire structure, the interlaced nanosheets form a three-dimensional network structure with larger sheet layer spacing, which is more conducive to the migration of electrons in the catalyst and the interlayer migration of hydroxide groups and water molecules during the OER process. As observed in the figure, the spacing of the nanosheets gradually decreased with the increase of Mo content. When Mo element is 20 wt%, the spacing of nanosheets decreased to about 1 μm, which is of benefit in increasing the active sites, and facilitates the adsorption and desorption of intermediates, which is good for improving the electrocatalytic hydrolysis process. As shown in Figure 4g, Ni, Co, Mo and O are uniformly distributed on the surface of the nanosheets, indicating that the surface composition of the NiCoMo_2_-LDH/NF electrocatalyst is relatively uniform, which ensures the stability of catalytic performance.

**Figure 5:**
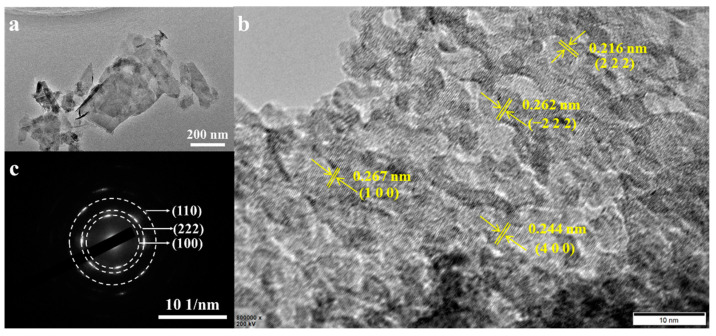
(**a**) TEM images, (**b**) HRTEM image and (**c**) SAED pattern of NiCoMo_2_-LDH/NF.

The structure of the NiCoMo_2_-LDH/NF nanosheets was further confirmed by the TEM analysis (Figure 5a). As shown in Figure 5a, the NiCoMo_2_-LDH/NF nanosheets can be observed clearly. The high-resolution TEM (HRTEM) image (Figure 5b) shows various kinds of lattice fringes with distances of 0.216, 0.244, 0.262 and 0.267 nm corresponding to the (222), (400) and (222) planes of CoMoO_4_ and (100) planes of Ni(OH)_2_, respectively. As shown in Figure 5c, the selected area electron diffraction (SAED) pattern shows (100) and (110) planes for Ni(OH)_2_, and (222) planes for CoMoO_4,_ indexed according to the reported X-ray diffraction (XRD) data.

### 3.2. Oxygen Evolution Reaction Performance

**Figure 6:**
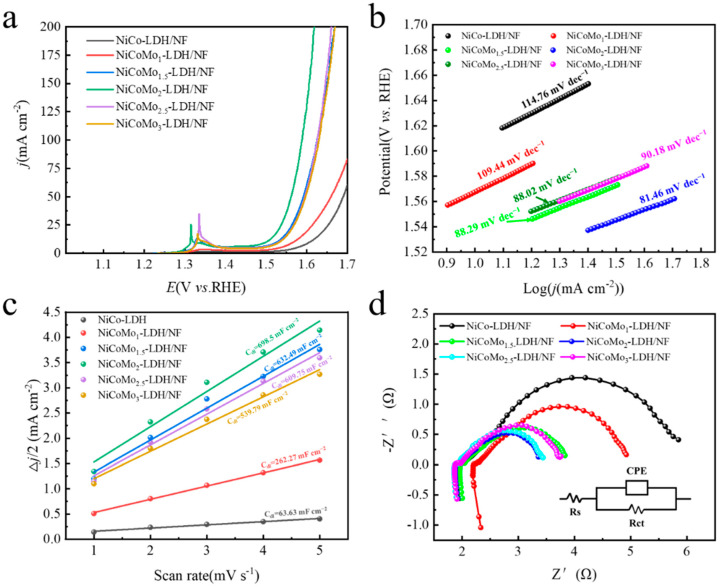
Polarization curves of Mo-doped electrode catalysts. (**a**) LSV curves of NiCoMo-LDH/NF in O_2_-saturated 1.0 M KOH solution at a scan rate of 5 mV s^−1^, (**b**) Tafel plot (overpotential vs. log current) derived from the corresponding polarization curves, (**c**) Plots showing the extraction of the double-layer capacitances allow the estimation of the electrochemically active surface area (ECSA), and (**d**) Nyquist plots recorded at overpotential of 1.56 V vs RHE.

OER performance of catalysts was studied in oxygen-saturated electrolytes by using different Mo-doped electrocatalysts as working electrodes. All catalysts were activated before testing to ensure stable catalyst performance. Electrochemical measurements of electrode catalysts with different Mo content are shown in Figure 6. Figure 6a shows the polarization curves of different Mo-doped electrocatalysts after iR-drop correction. The overpotential of NiCoMo_2_-LDH/NF nanosheets is 270 mV at a current density of 10 mA cm^−2^, which is significantly smaller than that of NiCo-LDH/NF (377.2 mV), NiCoMo_1_-LDH/NF (337.2 mV), NiCoMo_1.5_-LDH/NF (296.2 mV), NiCoMo_2.5_-LDH/NF (301.2 mV) and NiCoMo_3_-LDH/NF (303.2 mV) electrodes. Obviously, bimetallic NiCo-LDH/NF shows poorer OER activity than other ternary electrode materials. It can be seen that an appropriate amount of Mo doping can effectively reduce the overpotential and improve the OER performance.

In order to evaluate the OER kinetics of the catalyst, the Tafel slope is calculated according to the LSV curve from 1.0 to 1.7 V (Figure 6b). The linear regions of the Tafel plots are fitted to the Tafel equation: η = b log j + a, where η is the overpotential, b is the Tafel slope, and j is the current density. The Tafel slope of NiCoMo_2_-LDH/NF is 81.46 mV dec^−1^, which is much smaller than NiCo-LDH/NF 114.76 mV dec^−1^, NiCoMo_1_-LDH/NF 109.44 mV dec^−1^, NiCoMo_1.5_-LDH/NF 88.29 mV dec^−1^, NiCoMo_2.5_-LDH/NF 88.02 mV dec^−1^ and NiCoMo_3_-LDH/NF 90.18 mV dec^−1^. Obviously, among the electrocatalysts with different Mo-doped, NiCoMo_2_-LDH/NF shows a superior OER performance.

The electrochemical active surface area (ECSA) is directly proportional to the electrochemical double-layer capacitance (C_dl_). More exposed active sites can be provided for the electrocatalytic reaction when there is a large ECSA. ECSA is calculated as ECSA = C_dl_/C_s_, where C_dl_ is the electrochemical double-layer capacitance, and C_s_ is the specific capacitance of the electrode material plane between 20–60 μF cm_ECSA_^−2^ [30,31]. The scanning speed is 1–5 mV s^−1^. C_dl_ is measured by cyclic voltammetry (CV) in the range of 1.317 ~ 1.417 V vs. RHE. Figure 6c shows the plots of C_dl_ of electrocatalysts in current (∆ j/2 = (j_a_ − j_c_)/2 at 1.3672 V vs. RHE) against the scan; the C_dl_ of NiCoMo_2_-LDH/NF is 689.5 mF cm^−2^, which is larger than that of NiCo-LDH/NF (63.63 mF cm^−2^), NiCoMo_1_-LDH/NF (262.27 mF cm^−2^), NiCoMo_1.5_-LDH/NF (632.49 mF cm^−2^), NiCoMo_2.5_-LDH/NF (609.75 mF cm^−2^) and NiCoMo_3_-LDH/NF (539.79 mF cm^−2^). Obviously, the high C_dl_ of NiCoMo_2_-LDH/NF is attributable to the exposure of more active sites in the layered structure, which significantly enhances the OER performance of the electrode catalyst.

EIS reflects the dynamics of electrode and electrolyte surfaces. The charge transfer characteristics in the OER process at 1.56 V vs. RHE were analyzed. The corresponding solution resistance (R_s_) and charge transfer resistance (R_ct_) can be obtained by fitting the impedance diagram with the ZSimpWin software, where the R_ct_ value is related to the size of the semicircle radius in the impedance diagram, and the smaller the R_ct_ value the smaller the semicircle radius in the impedance diagram, and the faster the charge transfer rate at the electrocatalyst and electrolyte interface. EIS results are depicted in Figure 6d, accompanied by the equivalent circuit mode. The excellent OER performance of NiCoMo_2_-LDH/NF nanosheets is attributable to the rapid charge transfer, which can be seen in the smaller semicircle of the impedance diagram; the smaller the radius of the impedance diagram, the faster the charge transfer rate and the better the kinetic performance of the electrode catalyst. Fitting an equivalent circuit to the impedance plot shows that the solution resistance (R_s_) values of all catalysts are similar and smaller than 2.3 Ω. NiCoMo_2_-LDH/NF exhibits a much smaller R_ct_ (1.64 Ω) than NiCo-LDH/NF (4.50 Ω), NiCoMo_1_-LDH/NF (2.86 Ω), NiCoMo_1.5_-LDH/NF (2.08 Ω), NiCoMo_2.5_-LDH/NF (1.65 Ω) and NiCoMo_3_-LDH/NF (1.98 Ω). This indicates the faster electron transport kinetics of NiCoMo_2_-LDH/NF at the interface between electrodes.

**Figure 7:**
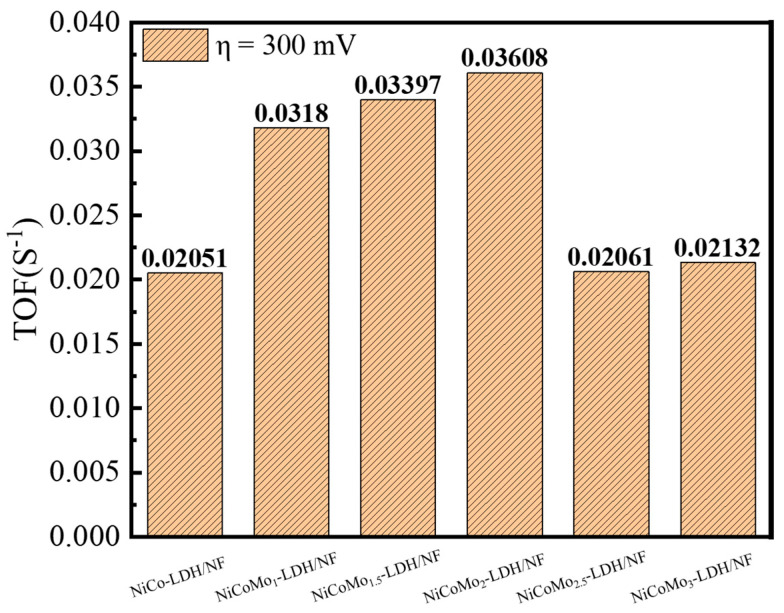
TOF values of different Mo-doped electrode catalysts at an overpotential of 300 mV.

The turnover frequency (TOF) of the catalyst can be used to reflect the intrinsic activity of the material. The TOF value can be calculated using the following equation: TOF = (jA)/(4Fm), where j is the current density measured by the LSV curve at overpotential of 300 mV, A is the area of the working electrode, F is the Faradic constant (96,485 C mol^−1^), and m is the number of active sites (mol) quantified by CV technique [32,33]. By measuring a series of CV curves within a potential range between 1.0072 to 1.8072 V vs. RHE, m can be obtained from the linear relationship between oxidation peak current and scan rate [33]. Meanwhile, it is assumed that under ideal conditions, all the surface active sites of the electrocatalyst are involved in the electrocatalytic reaction. Figure 7 shows TOF values of different Mo-doped electrocatalysts, the calculated TOF of NiCoMo_2_-LDH/NF is 0.03608 s^−1^ at an overpotential of 300 mV, which is higher than that of NiCo-LDH/NF (0.02051 s^−1^), NiCoMo_1_-LDH/NF (0.0318 s^−1^), NiCoMo_1.5_-LDH/NF (0.03397 s^−1^), NiCoMo_2.5_-LDH/NF (0.02061 s^−1^) and NiCoMo_3_-LDH/NF (0.02132 s^−1^). It is indicative that the intrinsic activity of NiCoMo_2_-LDH/NF electrocatalyst is higher than that of other samples, and the high electron conversion rate can quickly improve the OER reaction kinetics. More comparison of OER performance of different nickel- or cobalt-based electrocatalysts are shown in Table 1. As can be seen from Table 1, compared with monometallic catalysts, ion doping can significantly change the microscopic morphology of the catalysts, forming porous structures such as nanowires, nanosheets and hollow microcuboids in situ on the substrate surface, and the secondary pores grown in situ significantly increase the active surface area of the polymetallic catalysts, allowing the electrolyte and the catalyst to be in full contact, which is conducive to the electrocatalytic process and greatly reduces the overpotential of the system.

**Figure 8:**
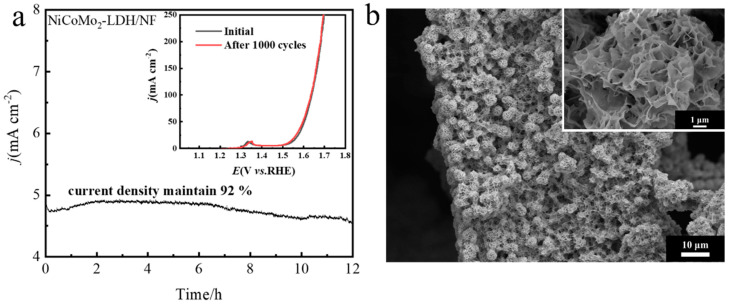
Stability test. (**a**) I-T curve of the NiCoMo_2_-LDH/NF electrocatalyst, initial LSV curve and LSV curve after 1000 CV cycles, and (**b**) Low magnification and high magnification inset SEM images after NiCoMo_2_-LDH/NF stability test.

Long-term stability is an important index for evaluating the practical application of electrocatalysts. The chronopotentiometry, LSV and CV were utilized to evaluate the electrocatalytic durability of a NiCoMo_2_-LDH/NF electrocatalyst for OER in 1.0 M KOH. As shown in Figure 8a, the I-t curve shows that NiCoMo_2_-LDH/NF still maintains excellent OER stability after a 12 h stability test in alkaline solution. NiCoMo_2_-LDH/NF was treated firstly via CV in a potential range of 0 to 0.7 V with a scan rate of 0.1 V s^−1^ for 1000 cyclesl; the LSV curves were recorded before and after the CV test. According to the SEM image after the stability test in Figure 8b, after a 12 h stability test, the NiCoMo_2_-LDH/NF electrocatalyst still maintained its inherent morphology, which also proves its excellent stability.

## 4. Conclusions

In summary, we have successfully synthesized a Mo-doped NiCoMo-LDH/NF nanoscale catalyst on NF substrate by using a simple one-step hydrothermal method. The NiCoMo_2_-LDH/NF nanosheet catalyst has superior OER performance and stability. In addition, we have achieved an overpotential of 270 mV by using NiCoMo-LDH/NF as an OER catalyst at a current density of 10 mA cm^−2^, and its Tafel slope can reach 81.46 mV dec^−1^, which is significantly better than other reported OER catalysts. It is found that Mo doping result in the transformation of NiCo-LDH/NF electrode catalysts from nano linear to nanosheets. The transformation contributes to improving the catalytic sites on the surface of the electrode catalyst and is conducive to the rapid transfer of electrons and the adsorption and desorption of intermediates. NiCoMo-LDH/NF nanosheet catalyst shows excellent electrocatalytic activity and stability, rich materials and low cost. Therefore, it may have a promising application in alkaline electrolytic water.

## Figures and Tables

**Table 1:** More comparison of OER performance of different nickel- or cobalt-based electrocatalysts (overpotentials η calculated by using the formula η = *E*_RHE_ − 1.23 V).

Catalyst	Current Density (mA cm^−2^)	Overpotential (mV)	Reference
Ni(OH)_2_	10	595	[34]
Co(OH)_2_ nanoflake/Ni foam	10	280	[35]
Co-Ni-B/NF	10	313	[36]
NiCo hydroxide	10	460	[37]
NiCo-LDH	10	367	[38]
NiCo-NS	10	334	[39]
NiCo_2_O_4_ hollow microcuboids	10	277	[40]
NiCo_2_S_4_@graphene	10	470	[41]
Ni_x_Co_2x_(OH)_6x_@eRG/NF	10	280	[42]
ZIF-67/CoNiAl-LDH/NF	10	303	[43]
(Ni_1−x_Co_1+x_Se_4_)MPs	10	320	[44]
NiCo-LDH nanosheets	10	290	[45]
Co(OH)_2_	10	360	[46]
NiCoMo_2_-LDH/NF	10	270	This work

## Data Availability

The data presented in this study are available on request from the corresponding author.
